# The Mechanism of Ajmaline and Thus Brugada Syndrome: Not Only the Sodium Channel!

**DOI:** 10.3389/fcvm.2021.782596

**Published:** 2021-12-23

**Authors:** Michelle M. Monasky, Emanuele Micaglio, Sara D'Imperio, Carlo Pappone

**Affiliations:** ^1^Arrhythmology Department, IRCCS Policlinico San Donato, San Donato Milanese, Milan, Italy; ^2^Vita-Salute San Raffaele University, Milan, Italy

**Keywords:** Brugada syndrome (BrS), ajmaline, arrhythmias, sudden cardiac death (SCD), sodium channel, potassium channel, calcium channel, mitochondria

## Abstract

Ajmaline is an anti-arrhythmic drug that is used to unmask the type-1 Brugada syndrome (BrS) electrocardiogram pattern to diagnose the syndrome. Thus, the disease is defined at its core as a particular response to this or other drugs. Ajmaline is usually described as a sodium-channel blocker, and most research into the mechanism of BrS has centered around this idea that the sodium channel is somehow impaired in BrS, and thus the genetics research has placed much emphasis on sodium channel gene mutations, especially the gene *SCN5A*, to the point that it has even been suggested that only the *SCN5A* gene should be screened in BrS patients. However, pathogenic rare variants in *SCN5A* are identified in only 20–30% of cases, and recent data indicates that *SCN5A* variants are actually, in many cases, prognostic rather than diagnostic, resulting in a more severe phenotype. Furthermore, the misconception by some that ajmaline only influences the sodium current is flawed, in that ajmaline actually acts additionally on potassium and calcium currents, as well as mitochondria and metabolic pathways. Clinical studies have implicated several candidate genes in BrS, encoding not only for sodium, potassium, and calcium channel proteins, but also for signaling-related, scaffolding-related, sarcomeric, and mitochondrial proteins. Thus, these proteins, as well as any proteins that act upon them, could prove absolutely relevant in the mechanism of BrS.

## Introduction

Ajmaline is used as a pharmacologic test to diagnose Brugada syndrome (BrS) and identify people who are at higher risk of developing life-threatening arrhythmias and sudden cardiac death. Many patients are ultimately implanted with an implantable cardioverter-defibrillator that can save their lives. The BrS is an inherited disease characterized by a coved-type ST-segment elevation in the right precordial leads (V_1_-V_3_) on the electrocardiogram (ECG). The true prevalence of BrS is unknown, since many people are asymptomatic. In fact, the syndrome may not even be suspected until an incidence of cardiac arrest. Certain “trigger situations,” such as fever, drug use, or consumption of alcohol or large meals can elicit the BrS ECG pattern (1). Since the systematic introduction of sodium-channel blockers to screen for the syndrome, the diagnosis, and thus the perceived incidence, of BrS has increased (2).

Sodium channel blockers, such as ajmaline, flecainide, or procainamide can be used to provoke the type-1 BrS ECG pattern, which is said to affirmatively diagnose the syndrome (3, 4). Thus, the disease is defined at its core as a particular response to these drugs. Some clinicians prefer the use of ajmaline, which appears to have a lower false negative rate, due to its higher sensitivity (5, 6). This higher sensitivity of ajmaline, compared to flecainide, may be due to flecainide's greater inhibition of I_to_, which then renders it less effective (5). Whole-cell patch clamp experiments demonstrated a reduced I_to_ total charge with an IC_50_ of 216 and 15.2 μM for ajmaline and flecainide, respectively, while sodium channel current was affected similarly by both drugs, as suggested by equivalent changes in QRS and PQ intervals (5). However, reports have cautioned about ajmaline's false positive rate, stating that a positive ajmaline test does not always mean that a patient has BrS (7–10). In fact, ajmaline metabolism is very complex (11) for several reasons relating to the liver metabolism, kidney metabolism, plasma proteins binding, and variability in the expression of ajmaline-metabolizing enzymes (12). Ajmaline undergoes some major metabolic pathways: mono- and di-hydroxylation of the benzene ring with subsequent O-methylation, reduction of the C-21, oxidation of both C-17 and C-21-hydroxyl function and N-oxidation (13). Consequently, one of the major genes controlling ajmaline metabolism is *CYP2D6*, encoding for a cytochrome C component. Thus, it is not surprising that patients harboring variants or even polymorphisms in the *CYP2D6* gene might display a different capability to metabolize ajmaline (14). To date, more than 70 allelic variants of the *CYP2D6* gene have been reported, and altered *CYP2D6* function has been associated with both adverse drug reactions and reduced drug efficacy (15). This is the main reason why poor metabolizer alleles can be important as a possible cause of false positivity during ajmaline challenge test.

Ajmaline challenges must be conducted in specialized centers due to the potential development of life-threatening ventricular arrhythmias, such as polymorphic ventricular tachycardia (VT) or ventricular fibrillation (VF) (16–18). Ajmaline infusion should be done carefully, stopping as soon as the result is positive or when QRS broadens to ≥130% of baseline or frequent pre-mature ventricular complexes occur (17, 19, 20).

Ajmaline is usually described as a sodium-channel blocker (3), and most research into the mechanism of BrS has centered around this idea that the sodium channel is somehow impaired in BrS (21, 22), and thus the genetics research has placed much emphasis on sodium channel gene mutations, especially the gene *SCN5A*, whereas systematic studies on other genes are lacking (23). The research up until this point has focused so much on the *SCN5A* gene that it has even been suggested that only the *SCN5A* gene should be screened in BrS patients (23), something that has been hotly debated (24–26), as many argue that research is needed to understand the possible role of several other genes in this disease (27–32). However, pathogenic rare variants in *SCN5A* are identified in only 20–30% of ajmaline-positive cases (33–36), and recent data indicates that mutations in *SCN5A* are actually, in many cases, prognostic rather than diagnostic, resulting in a more severe phenotype (26, 35, 37–39). Furthermore, the misconception by some that ajmaline only influences the sodium current, and thus sodium channels should be the only channels of interest in BrS, is flawed, in that ajmaline actually acts additionally on potassium and calcium currents, as well as mitochondria and metabolic pathways. Thus, potassium channels, calcium channels, mitochondrial proteins, and metabolic pathway proteins, or factors that act upon these proteins, could prove absolutely relevant, as their function is directly influenced by the very drug that is used to diagnose the disease in the first place.

## Multiple Binding Sites of Ajmaline On Na^+^, K^+^, and Ca^2+^ Channels

Ajmaline has multiple sites of action, including sodium, potassium, and calcium channels. Plant alkaloids, including ajmaline, act on at least six receptor sites on voltage-gated Na^+^ channels (40). In single intact amphibian skeletal muscle fibers, it appeared that ajmaline has multiple sites of action, including the positively charged S4 voltage-sensing segment of Na^+^ and K^+^ channels (40). However, ajmaline also blocks channels that do not have a voltage sensor (e.g., K_ATP_) (40).

In human embryonic kidney (HEK) cells, ajmaline has an inhibitory effect on human ether a-go-go related gene (HERG) potassium channels in the open, but not in the closed states, and probably binds at aromatic residues Tyr-652 and Phe-656 in the channel pore cavity (41). The inhibitory effect was stronger at higher frequencies (41). Ajmaline is an open channel inhibitor at therapeutic concentrations of cardiac potassium K_V_1.5 and K_V_4.3 channels, responsible for cardiac I_Kur_ and I_to_ current, respectively (42). Ajmaline potently blocks glibenclamide-sensitive K^+^ channels in *Xenopus* oocytes in a concentration-dependent manner (43). There is an effect of ajmaline on the inhibition of K^+^ outflow from rat liver mitochondria (44). In rat right ventricular myocytes, the decreased amplitude and time integral of I_to_ by ajmaline is dependent on concentration, but not frequency or use (45). In rat right ventricular myocytes, ajmaline blocks the transient outward potassium current (I_to_) when the channel is in the open state and there is fast recovery from the block at resting voltage (45).

Whole cell patch clamp technique used to determine the effect of ajmaline on action potential (AP) and ionic current components in rat right ventricular myocytes demonstrated an inhibitory effect on sodium current (I_Na_), L-type calcium current (I_Ca−L_), transient outward potassium current (I_to_), the current measured at the end of 300 ms depolarizing pulse (I_K,end_), and ATP-sensitive potassium current [I_K(ATP)_] (46). The inhibition of I_Na_ causes both the decreased rate of rise of depolarizing phase and the lowered amplitude of AP (46). Additionally, I_to_ inhibition was responsible for AP prolongation after ajmaline administration (46). In isolated guinea pig ventricular cardiomyocytes, ajmaline suppressed calcium currents (I_Ca_) in a dose-dependent manner without affecting the steady-state inactivation kinetics and the voltage dependency of the current-voltage relationship, inhibited inwardly rectifying potassium current (I_K1_), and decreased the delayed rectifier potassium current (I_K_) without altering the activation or deactivation time courses (47). A study recording intracellular action potentials and transmural ECG in canine RV wedge preparations suggested that combined sodium and calcium channel block may be more effective than sodium channel block alone in unmasking the BrS pattern (48). The study used terfenadine to block both sodium and calcium current, which resulted in the loss of the epicardial AP dome, ST segment elevation, phase 2 reentry, and spontaneous polymorphic VT/VF (48). This effect of terfenadine was normalized with 4-aminopyridine, which inhibits I_to_ (48). The drugs flecainide, ajmaline, and procainamide alone did not generate polymorphic VT, but they did so together with the calcium channel blocker verapamil (48).

N-propyl ajmaline (NPA) is the quaternary derivative of ajmaline. The permanently charged NPA and protonated ajmaline both act mainly with open channels, while unprotonated ajmaline acts mainly on inactivated Na^+^ channels in frog myelinated fibers (49). In frog myelinated fibers, sodium and potassium currents are inhibited by ajmaline and NPA, for sodium in both directions, but for potassium, only the outward potassium current, not the inward potassium current (49). The location of the binding sites have been suggested to be in the inner mouths of Na^+^ and K^+^ channels (49). In voltage clamp experiments using frog nodes of Ranvier, the binding site for NPA has been described to be located in the inner mouth of the Na^+^ channels, and it becomes available to the charged blocker (NPA) only after opening of the activation gate (50). NPA in enzymatically isolated cells of adult rats inhibits I_Na_ due to a voltage-dependent interaction with open Na^+^ channels, and NPA has similar blocking effects on Na^+^ channels in myocardial cells and nerve fibers (51).

## Genetics of Channels Implicated by Functional Studies

**Figure 1:**
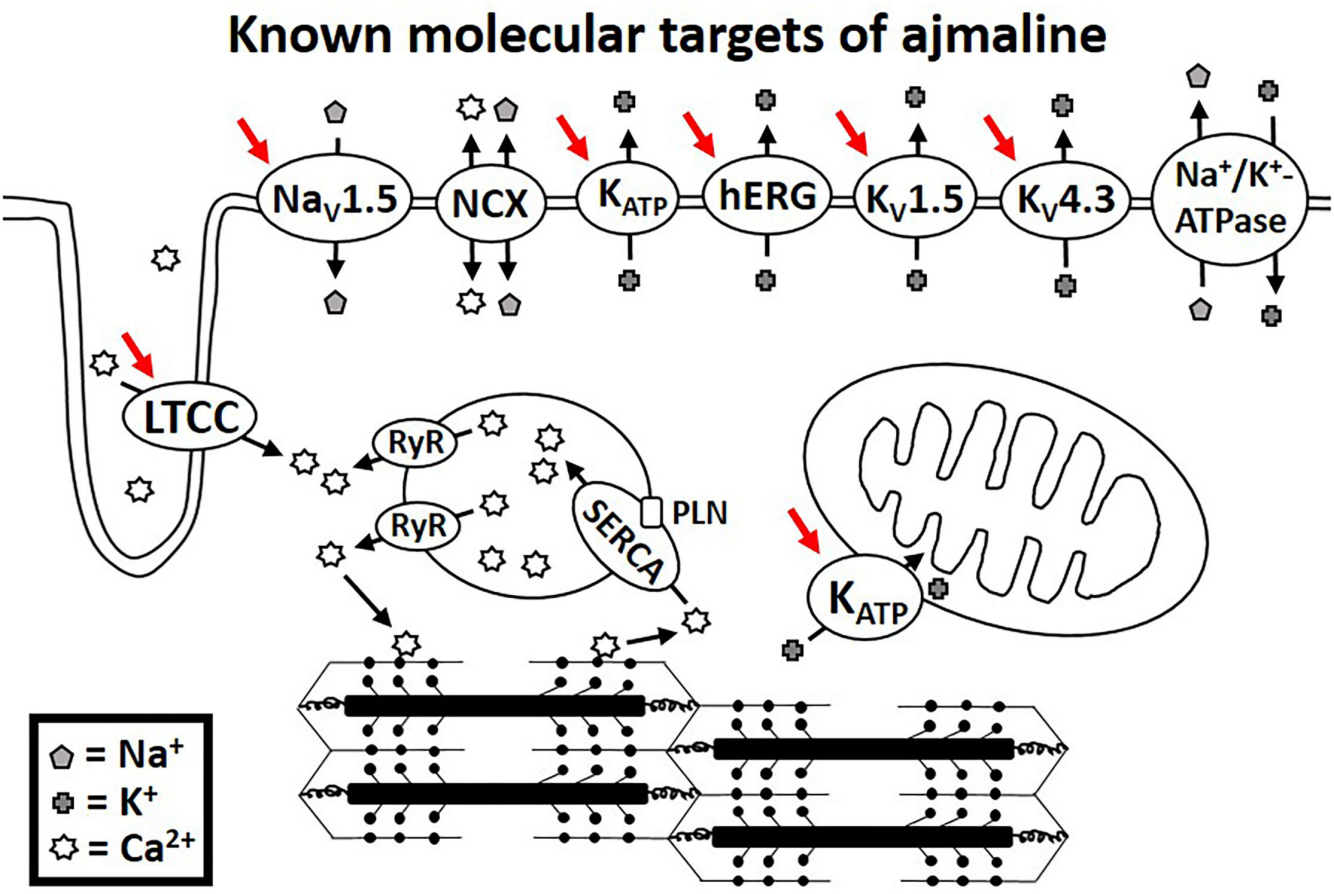
Molecular targets of ajmaline as suggested by functional studies indicated by red arrows.

Functional studies have identified several molecular targets of ajmaline. Many of these molecular targets are encoded for by genes that have been associated with BrS in clinical studies. Table 1 lists the known molecular targets of ajmaline and their related genes. Figure 1 shows a schematic of ajmaline targets in the cell, as demonstrated by functional studies.

**Table 1:** Known molecular targets of ajmaline and potential genes that they implicate.

**Protein or current described in functional studies targeted by ajmaline**	**Examples of genes that these targets implicate (52)**
Sodium channel current (I_Na_) (40, 46)	*SCN5A, SCN10A, SCN1B, SCN2B, SCN3B, SCN4A*
Potassium channel current (I_K_) (46)	*KCNA4, KCNE4*
ATP-sensitive potassium channel (K_ATP_) (40, 46)	*ABCC8, ABCC9, KCNJ1, KCNJ5, KCNJ8, KCNJ11*
human ether a-go-go related gene (HERG) potassium channels (41)	*hERG (KCNH2)*
K_V_1.5 channels, responsible for cardiac I_Kur_ (42)	*KCNA5*
K^+^ outflow from mitochondria (mitoK_ATP_) (44)	Formed by 5 components (53): • Mitochondrial ATP-binding cassette protein 1 (mABC1): *ABCB8* • Phosphate carrier: *MPCD, SLC34A1, SLC17A1, SLC17A7, SLC17A6, SLC25A26, SLC25A3, SLC25A25, SLC37A4, SLC25A23* • Adenine nucleotide translocator: *SLC25A4, SLC25A5, SLC25A6, SLC25A31, SLC25A6* • ATP synthase: *ATP5PF, ATP5F1C, ATP5F1B, ATP5F1D, ATP5F1A, ATP5ME, MC5DN2, ATP5PO, ATP5G1, ATP5G2* • Succinate Dehydrogenase: *SDHC, SDHB, SDHA, SDHD, SDHAF2, SDHAF4, SDHAF1* (2021)
K_V_4.3 channels and outward potassium current (I_to_) (42, 45)	*KCND3*
L-type calcium current (I_Ca−L_) (46)	*CACNA1C, CACNB2*
inwardly rectifying potassium current (I_K1_) (47)	*KCNJ2, KCNJ5, KCNJ8*
delayed rectifier potassium current (I_K_) (47)	*KCNS3, KCNS1, KCNS2*

### A Unique Role for Calcium: Excitation-Contraction Coupling in Brugada Syndrome

Calcium signaling is responsible for connecting the electrical signaling of the cell to the mechanical force of contraction and relaxation of the sarcomeric proteins. Thus, calcium imbalances in the cell could result in alterations to the force production. In porcine epicardial shavings, excitation failure by current-to-load mismatch was shown to cause ST segment elevation modulated by I_to_ and I_CaL_ (54). A study by Biamino et al. demonstrated a relaxing effect of ajmaline on vascular smooth muscle using aortic helical strips, attributing the effect possibly to a reduction in Ca^2+^ and probably Na^+^ conductance (55). In BrS patients, ajmaline administration results in a decrease of right ventricular ejection fraction and minimum principal strain in the right ventricular outflow tract and right ventricular anterior wall (56, 57). In fact, it has been previously suggested that the electromechanical coupling in BrS, including calcium handling and sarcomeric alterations, should be investigated (28, 57). Reduced intracellular calcium, which may result in a reduction of force production, has been proposed as a possible mechanism in BrS (8, 28, 58, 59). Additionally, administration of pharmaceuticals that act on outer cell membrane receptors can result in signaling changes within the cell (60, 61). It would be interesting to see in future studies whether ajmaline affects intracellular processes, such as signaling pathways that lead to post-translational modifications, affecting various proteins, such as those located in the sarcoplasmic reticulum or the myofilaments.

## Genetics of Channels Implicated by Clinical Studies

The genetics of BrS remains a hotly debated subject. More than 20 genes are currently included in diagnostic genetic testing panels, previously reviewed in detail elsewhere (32), although the significance of variants in all but the *SCN5A* gene are disputed, since most studies to-date have focused on understanding better variants in the *SCN5A* gene, while studies on the other genes are generally lacking (23). However, pathogenic rare variants in *SCN5A* are identified in only 20–30% of ajmaline-positive cases (33–36), and recent data indicates that mutations in *SCN5A* are actually, in many cases, prognostic rather than diagnostic, resulting in a more severe phenotype (26, 35, 37–39). Several important studies of other genes are now available, and more are needed to better understand the mechanism of ajmaline in provoking the type-1 BrS ECG pattern.

Sodium channel-related genes other than *SCN5A* that have been previously implicated in BrS, and they include *SCN10A, SCN1B, SCN2B, SCN3B, SCN4B, RANGRF* (*MOG1*), and *GPD1L*. Potassium-related genes previously associated in BrS include *KCND2, KCND3, KCNE1, KCNE2, KCNE3, KCNE5, KCNH2, KCNJ2, KCNJ5, KCNJ8, KCNQ1, ABCC9*, and *HCN4*, while calcium-related genes previously described in BrS include *CACNA1C, CACNA2D1, CACNB2, RYR2*, and *TRPM4* (32, 62). In addition, the gene *PKP2* has been associated with BrS, and studies have shown a relationship between *PKP2* and both sodium and potassium channels. For example, in a study by Cerrone et al., loss of PKP2 caused decreased I_Na_ and Na_V_1.5 (63). Hong et al. demonstrated an interaction between PKP2 and K_ATP_ channels in rat heart (64).

Sarcomeric properties have been directly linked to arrythmogenic sudden death (61, 65), and variants in myofilament genes, including *TPM1* and *MYBPC3*, have been found in BrS patients (27, 66, 67). Several other genes, encoding signaling and scaffolding proteins, including *AKAP9, ANK2, CASQ2, CAV3, CBL, DSC2, DSG2, DSP, FGF12, HEY2, JUP, LMNA, LRRC10, NOS1AP, SEMA3A, SLMAP, SNTA1*, and *TMEM43*, have been suggested as candidate genes (32, 62, 68). The function of proteins that are affected by protein kinase A or reactive oxygen species (ROS), such as the protein products of many of the genes listed above, may be altered by changes in mitochondrial function, which is responsible for ATP and ROS production (26). In fact, studies have implicated a direct role for mitochondria in BrS, specifically, severe cases have been associated with a particular mitochondrial DNA (mtDNA) allelic combination and a high number of mtDNA single nucleotide polymorphisms (69, 70), and a role for mitochondrial transfer RNA genes has been suggested (71). Thus, in addition to *SCN5A*, various other genes have been suggested to have a role in BrS, including other sodium channel-related genes, as well as several potassium-related, calcium-related, signaling-related, scaffolding-related, sarcomeric, and mitochondrial genes, consistent with the identified molecular targets of the ajmaline drug used to unmask and diagnosis the syndrome.

Although it is generally agreed that variants in the *SCN5A* gene are involved in BrS, it is important to think of variants even within this gene as individual variants with specific effects, rather than thinking of all *SCN5A* variants collectively, as some may be benign, while others pathogenic (26). Along these lines, several studies have sought to understand the effect of specific *SCN5A* variants (37, 72–80). It has been recently suggested that variants in the *SCN5A* gene may actually be prognostic, rather than diagnostic (35, 38, 39).

Studies to better understand the role of variants in each of the above-mentioned genes will be an important area of future research. A recent study by Di Mauro et al. demonstrated an important role for *CACNA1C* (31), highlighting the importance of functional studies of genes that may be involved in BrS, but for which we currently lack the proof (81). Recent studies have also focused on the roles of the genes *SCN10A* and *HEY2* in BrS (29, 82). However, much work remains to be done before we can understand the role of each of the protein products of these genes, as well as the role of the proteins that signal to them and alter their function. Currently, the understanding of genetics in BrS is in its infancy, and genetic testing alone should not be used for diagnostic purposes, but rather, diagnosis of BrS should be based upon an arrhythmological examination by a specialized cardiologist (26). The presence of a variant in the *SCN5A* gene, however, may be relevant for prognostic purposes (35, 38).

## Limitations and Future Studies

Most of the studies to better understand the mechanism of ajmaline have been performed in cellular models using non-cardiomyocyte cell types or in animal models that are sometimes not even mammalian. While these models give us some insight, each model comes with its own set of advantages and limitations (83). The functional studies performed to-date indicate that ajmaline does not act solely on sodium channels and suggests that clinical genetic testing could be expanded for research purposes to include, for example, genes that encode for potassium and calcium channels. Thus, the mechanism of BrS could be researched from also this clinical direction. Regarding future functional studies, it would be interesting to quantify ajmaline signaling to, and effects on, particular sodium, potassium, and calcium channels and the resulting effect of sodium, potassium, and calcium handling, to ultimately understand the mechanism behind the altered ECG.

## Conclusion

The misconception by some that ajmaline only influences the sodium current, and thus sodium channels should be the only channels of interest in BrS, is flawed, in that ajmaline actually acts additionally on potassium and calcium currents, as well as mitochondria and metabolic pathways. Clinical studies have implicated several candidate genes in BrS, encoding not only for sodium, potassium, and calcium channel proteins, but also for signaling-related, scaffolding-related, sarcomeric, and mitochondrial proteins. Thus, these proteins, as well as any proteins that act upon them, could prove absolutely relevant in the mechanism of BrS.

## Author Contributions

MM: conceptualization and writing—original draft preparation. MM, EM, and SD'I: literature search and writing—draft revision. CP: funding acquisition. MM, EM, SD'I, and CP: reviewed and provided comments. All authors have read and agreed to the published version of the manuscript.

## Funding

This study was partially supported by Ricerca Corrente funding from Italian Ministry of Health to IRCCS Policlinico San Donato. The funders had no role in the design of the study; in the collection, analyses, or interpretation of data; in the writing of the manuscript, or in the decision to publish the results.

## Conflict of Interest

The authors declare that the research was conducted in the absence of any commercial or financial relationships that could be construed as a potential conflict of interest.

## Publisher's Note

All claims expressed in this article are solely those of the authors and do not necessarily represent those of their affiliated organizations, or those of the publisher, the editors and the reviewers. Any product that may be evaluated in this article, or claim that may be made by its manufacturer, is not guaranteed or endorsed by the publisher.

## Abbreviations

AP: action potential
BrS: Brugada syndrome
Ca^2+^: calcium
ECG: electrocardiogram
HEK: human embryonic kidney
HERG: human ether a-go-go related gene
I_Ca−L_: L-type calcium current
I_K_: delayed rectifier potassium current
I_K1_: inwardly rectifying potassium current
I_K(ATP)_: ATP-sensitive potassium current
I_K,end_: the current measured at the end of 300 ms depolarizing pulse
I_Kur_: ultrarapid outward potassium current
I_Na_: sodium current
I_to_: transient outward potassium current
K^+^: potassium
K_ATP_: ATP-sensitive potassium channel
Na^+^: sodium
NCX: sodium-calcium exchanger
NPA: N-propyl ajmaline, a quaternary derivative of ajmaline
PVCs: pre-mature ventricular complexes
RV: right ventricle
Vas: ventricular arrhythmias
VF: ventricular fibrillation
VT: ventricular tachycardia.
